# Supplementary material for: Higher Risk of Acute Respiratory Distress Syndrome and Risk Factors among Patients with COVID-19: A Systematic Review, Meta-Analysis and Meta-Regression
Source: Int J Environ Res Public Health. 2022 Nov 16;19(22):15125. doi: 10.3390/ijerph192215125 (PMC9690625; doi:10.3390/ijerph192215125)
Supplement: Supplementary file 1 [file ijerph-19-15125-s001.zip › ijerph-1985326-supplementary.pdf]

Supplemental Table S1. The Joanna Briggs Institute score for quality of evidence for prevalence studies

Topic / Authors (year)	Was the Sample frame appropriate to address the target population?	Were study participants sampled appropriately?	Was the sample size adequate?	Were the study subjects and the setting described in detail?	Was the data analysis conducted with sufficient coverage of the identified sample?	Were valid methods used for the identification of the condition?	Was the condition measured in a standardized, reliable way for all participants?	Was there An appropriate statistical analysis?	Was the response rate adequate, and if not, was the low response rate managed appropriately?	Overall appraisal: Include Exclude seek further info Total met criteria
Dreher, M. (2020)	√	√	√	√	√	√	√	√	√	9
Wang, A. (2020)	√	√	√	√	√	√	√	√	√	9
Wu, C. (2020)	√	√	√	√	U	√	√	√	√	8
El-Solh, A. A. (2021)	√	√	√	√	U	√	√	√	√	9
Hu, X. S.	√	√	√	√	√	√	√	√	√	9

(2021)										
Mizera, L. (2021)	√	√	√	√	√	√	U	√	√	8
Sehgal, T. (2021)	√	√	√	√	√	√	√	√	√	9
Seo, J. W. (2021)	√	√	√	√	√	√	U	√	√	8
Singhal, L. (2021)	√	√	√	√	U	√	√	√	√	8
Vassiliou, A. G. (2021)	√	√	√	√	√	√	√	√	√	9
Xu, W. (2021)	√	√	√	√	√	√	√	√	√	9
Gujski, M. (2022)	√	√	√	√	√	√	√	√	√	9

Note: √ = Yes, X = No, U = Unclear, N/A = Not applicable
